# Freezing of gait associated with a corpus callosum lesion

**DOI:** 10.1186/s40734-016-0030-2

**Published:** 2016-01-29

**Authors:** Marian L. Dale, Martina Mancini, Carolin Curtze, Fay B. Horak, Brett W. Fling

**Affiliations:** Department of Neurology, Oregon Health & Science University, 3181 SW Sam Jackson Road, 97239 Portland, OR USA; VA Portland Health Care System, Parkinson’s Disease Research, Education and Clinical Center, P3-PADRECC, PO Box 1034, 97207 Portland, OR USA

**Keywords:** Motor control, Diffusion tensor imaging

## Abstract

Freezing of gait (FoG) is a debilitating feature of Parkinson’s disease and other parkinsonian disorders. This case demonstrates a variant of freezing of gait in a non-parkinsonian patient with a lesion of the anterior corpus callosum. The freezing improved with increased upper extremity sensory input, suggesting that compensatory circuits for use of somatosensory inputs from the arms to postural and locomotor centers were intact.

**Electronic supplementary material:**

The online version of this article (doi:10.1186/s40734-016-0030-2) contains supplementary material, which is available to authorized users.

## Background

Freezing of gait (FoG) has been defined as a brief, episodic absence or marked reduction of forward progression of the feet despite the intention to walk [1]. FoG impairs mobility, causes falls, and reduces quality of life in advanced Parkinson’s disease (PD) and other parkinsonian syndromes. This case demonstrates a variant of freezing of gait in a patient who does not have Parkinson’s disease but has a lesion of the anterior corpus callosum.

## Case presentation

A 78 year-old woman complained of a gradually progressive gait abnormality, beginning with difficulty initiating steps after rising from a chair. Her symptoms progressed steadily over four years, to include shuffling during forward walking and FoG with both gait initiation and passage through doorways. She felt that her freezing was significantly improved by the use of a rolling walker. She noticed that her right leg tended to freeze more than the left, but denied resting tremor or stiffness. She also denied falls (including backward), other coordination difficulties, autonomic complaints, and numbness or weakness. Upper limb functioning was normal. A previous trial of levodopa (up to 800 mg daily) did not alleviate her symptoms. Medical history included non-insulin dependent diabetes mellitus, uncontrolled hyperlipidemia, and remote 3 pack-year cigarette exposure. She denied exposure to neuroleptics.

Examination revealed an oriented woman with normal language. Concentration was intact to complex backward spelling; executive function was intact to bedside Luria sequence testing. More detailed testing, however, revealed visuospatial and executive function deficits (clock drawing, box drawing, and Trails Part B), with a MOCA score of 22/30. Cranial nerves were normal. Tone and strength were normal. Resting tremor and bradykinesia were absent, including in the lower extremities. Proprioception was intact in the toes; coordination was normal. The right patellar reflex was brisk with crossed adduction; other reflexes were normal. Unassisted gait was wide based with freezing upon standing and prominent freezing with both straight walking and turning (see Additional file 1: video segment 1). The right foot was more affected by freezing with a pattern consisting of several small right steps with the right foot followed by one l step with the left. Walking with trekking poles resulted in dramatic improvement of gait and lessening of freezing (see Additional file 1: video segment 2). Despite significant gait impairments, standing balance in quiet stance was intact with normal postural sway, even on a foam surface.

### Gait neurophysiology

**Fig. 1:**
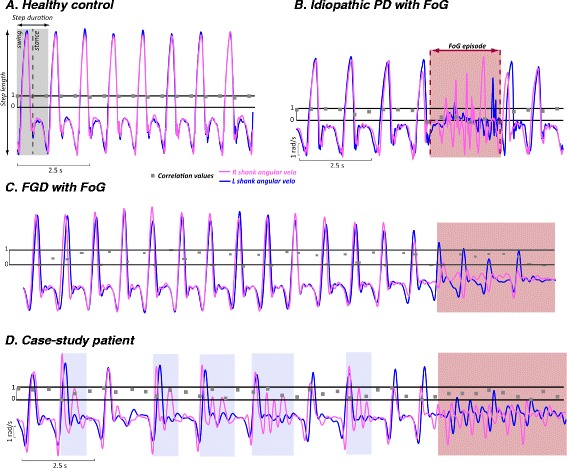
Gait neurophysiology. Legend: Cross-Covariance and Correlation of Ankle Angular Velocity in a healthy control (**a**), an idiopathic PD patient with a FoG episode, red shaded area (**b**), a representative vascular PD subject with a FoG episode (**c**), and in this patient (**d**). In order to overlap the right and left angular velocity signals, we calculated the lag between the right and left mid-swing peak by a cross-covariance between the two signals. Once the lag was determined, we shifted the signals by that amount so that the right and left shank angular velocities are synchronized. Then, we calculated the correlation between the two signals for each 0.5 s (fc = 200 samples/s). This approach allowed us to visually observe the strong asymmetry between the right and left leg during straight walking in this specific patient. The red shaded area in 1b, c, and d highlight FoG episodes during turning

Objective measures of gait from body-worn inertial sensors [2] showed significant asymmetry of right and left lower leg angular velocity during forward walking, caused by a peculiar pattern of multiple, small right steps followed by a singular left step (“floor scanning”). This phenomenon was only related to this case, and not seen in healthy control subjects, idiopathic PD or vascular parkinsonism (Fig. 1d vs. a, b, and c). The areas shaded in light blue (Fig. 1a vs. d) highlight this interesting non-reciprocal, floor scanning gait pattern. The red shaded areas highlight separate FoG episodes during turning.

### Neuroradiological data

**Fig. 2:**
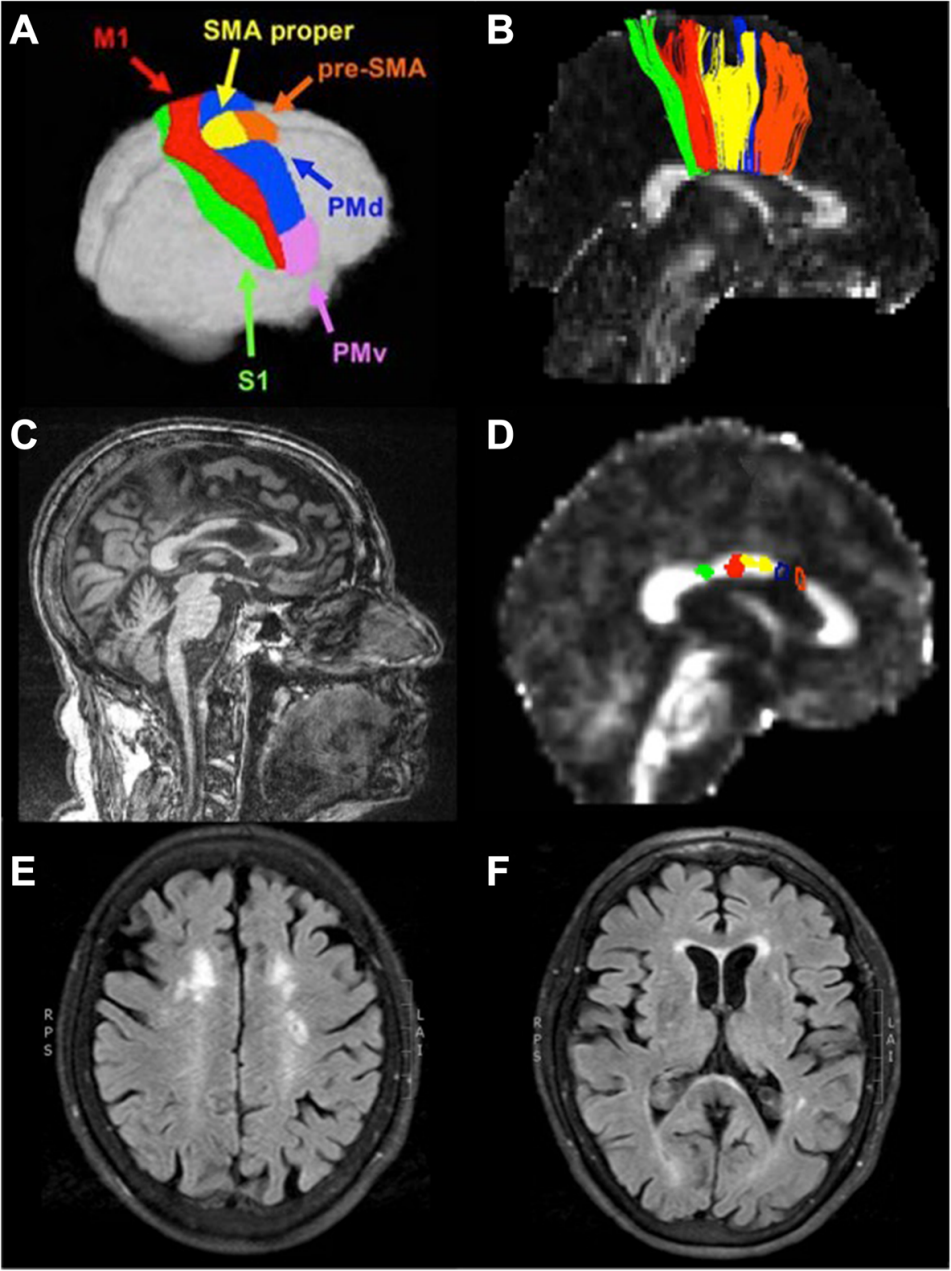
Diffusion tensor imaging: interhemispheric fiber tracts connecting principal, homologous sensorimotor cortical regions. Legend: **a** The Human Motor Area Template as defined by Mayka et al., 2006. **b** Interhemispheric fiber tracts from a healthy young adult, color-coded to match the cortical regions shown in panel A, taken from Fling et al., 2013. **c** Patient’s T1-weighted structural MRI showing an infarct within the anterior body of the corpus callosum. **d** Patient’s fractional anisotropy map, derived from diffusion weighted data. Fiber tracts were readily identified connecting the S1, M1, and SMA. No fiber tracts connecting either the PMd or the pre-SMA were identified; the blue and orange outlines demonstrate the location in the callosum where these fiber tracts would be expected to cross. **e & f** White matter hyperintensities within bilateral anterior periventricular regions. S1 = primary somatosensory cortex; M1 = primary motor cortex; SMA = supplementary motor area; PMd = dorsal premotor cortex; PMv = ventral premotor cortex

MRI of the brain showed a lesion in the anterior corpus callosum (Fig. 2c). It restricted diffusion but did not have an apparent diffusion coefficient (ADC) correlate to suggest acute infarct; nor did it enhance. Additional subcortical white matter changes were present in anterior periventricular and centrum semiovale regions, and were slightly more marked on the left (Fig. 2e and f). MRI of the thoracic spine showed mild degenerative changes only. A DaT scan was not performed. Probabilistic tractography derived from diffusion tensor imaging revealed interhemispheric fiber loss in regions of the corpus callosum responsible for connecting the dorsal premotor (PMd) and pre-supplementary motor areas (pre-SMA) (Fig. 2d). For representative control data, see Fig. 2a and b.

## Discussion

This case of freezing of gait (FoG) demonstrates several features not associated with FoG in idiopathic PD. There was no evidence of global parkinsonism or of lower extremity rigidity or bradykinesia. Asymmetric steps and freezing can be seen in idiopathic Parkinson’s disease or vascular parkinsonism, but the degree of asymmetry of lower extremity freezing with floor scanning was greater in this case [5]. In fact, this cautious, floor scanning strategy was only seen in this present case and not in the representative case of vascular PD with FoG, as reported in Fig. 1, where for each right step there is a single left step. In addition, the gait pattern of the vascular PD subject with FoG was very similar to the idiopathic PD case (both show a similar increase in gait asymmetry compared to a healthy control subject, but less asymmetry than the present case). This example of FoG shows near complete resolution with the use of trekking poles or a rolling walker. Parkinsonian freezing is often alleviated by the use of a rolling walker, but sometimes to a lesser degree, perhaps due to underlying bradykinesia. Furthermore, this patient’s balance was within normal range on foam surface testing, suggesting that balance deficits were not the primary reason for the improvement with upper extremity support.

We postulate that this patient has impaired coupling of bilateral leg movements, principally as a result of compromised callosal connections between the right and left pre-SMA (Fig. 2d). While damage to the frontal lobes caused by tumors, trauma, or stroke is known to cause gait abnormalities [1], lesions of the corpus callosum are not traditionally linked to FoG. However, recent work has shown that structural deficits in the genu of the corpus callosum are implicated in vascular parkinsonism [6, 7]. The current results support a growing body of literature demonstrating the importance of frontal and prefrontal cortical areas in motor control [8]. This case provides further evidence for the association of FoG and disruption of white matter pathways through the anterior corpus callosum, particularly highlighting the importance of interhemispheric pre-SMA connections.

Higher-level motor structures in the brain, including the SMA, PMd, and pre-SMA, provide input to the primary motor cortex to coordinate and control movement. For example, we have recently shown that the SMA is critical for control of anticipatory postural adjustments that shift the center of mass prior to stepping [9].

Anticipatory postural adjustments are not as necessary when there is external contact, such as proprioceptive information from the upper extremities [10], and this subject showed near complete resolution of her gait disorder with the use of trekking poles. The profound benefit of touching surfaces on her gait pattern suggests that compensatory circuits for use of somatosensory inputs from the arms to the postural and locomotor centers were intact in this patient. In addition, the upper extremity somatosensory input likely alleviated anxiety, a well recognized exacerbating factor for freezing of gait, related to both situational triggers and a fear of falling [11]. Finally, compensatory circuits for visually guided movements may have been strengthened in this case by the use of trekking poles [12], though a previous lack of improvement with other visual cues such as stepping over lines or the examiner’s foot argues against this. The even greater improvement in freezing after instruction in the proper use of trekking poles using alternating arm swing (see end of video) argues for the importance of compensatory circuits for interhemispheric motor control in freezing of gait.

We speculate that the callosal lesion in this case represents insidious leukoaraisosis due to chronic, uncontrolled diabetes mellitus and hyperlipidemia. There was no ADC correlate to suggest acute infarct at the time of imaging, and the history was one of more progressive gait dysfunction. The lack of enhancement argues against high-grade glioma. Though low-grade glioma is possible, the advanced age of the patient and lack of additional progression at subsequent visits make a neoplastic etiology unlikely.

The presentation was not suggestive of other neurodegenerative or inflammatory disorders. The lack of development of vertical saccade abnormalities or backward falls over four years argues against this being a case of pure akinesia related to progressive supranuclear palsy. The history was progressive rather than episodic, arguing against relapsing multiple sclerosis etiology, though neuroinflammation cannot be completely ruled out. We did not perform CSF analysis or additional neuropsychiatric testing. Though the etiology of this patient’s gait dysfunction was likely chronic microvascular damage, we argue that frontal gait disorder is a more applicable term than vascular parkinsonism, especially given the remarkable lack of lower body parkinsonism in this case.

## Conclusion

In sum, this case highlights the importance of the anterior corpus callosum in freezing of gait. Subcortical white matter changes present in the bilateral anterior periventricular and centrum semiovale regions may have also contributed to gait dysfunction in this patient, but the commissural callosal lesion is certainly the most prominent on imaging. We assert that this case exemplifies the importance of anterior callosal commissural pathways in the wide spectrum of frontal gait disorders.

## Consent

Written informed consent was obtained from the patient for publication of this case report and any accompanying images. A copy of the written consent is available for review by the Editor-in-Chief of this journal. This work was approved by the Institutional Review Board at OHSU, IRB# 4131.

## Additional file

Additional file 1: Segment 1: Unassisted gait was wide based with freezing upon standing and prominent freezing withboth straight walking and turning. The right foot was more affected by freezing with a pattern consisting ofseveral small right steps with the right foot followed by one l step with the left. Segment 2: Walking withtrekking poles resulted in dramatic improvement of gait and lessening of freezing.(M4V 4950 kb)

## Abbreviations

ADC: apparent diffusion coefficient
DaT: dopamine transporter
FoG: freezing of gait
M1: primary motor cortex
MOCA: Montreal Cognitive Assessment
PD: Parkinson’s Disease
PMd: dorsal premotor area
S1: primary somatosensory cortex
SMA: supplementary motor area

## References

[1] Nutt JG, Bloem BR, Giladi N (2011). Freezing of gait: moving forward on a mysterious clinical phenomenon. Lancet Neurol.

[2] Mancini M, King L, Salarian A, Holmstrom L, McNames J, Horak F (2012). Mobility lab to assess balance and gait with synchronized body-worn sensors. J Bioeng Biomed Sci.

[3] Mayka MA, Corcos DM, Leurgans SE, Vaillancourt DE (2006). Three-dimensional locations and boundaries of motor and premotor cortices as defined by functional brain imaging: a meta-analysis. Neuroimage.

[4] Fling BW, Cohen RG, Mancini M, Nutt JG, Fair DA, Horak FB (2013). Asymmetric pedunculopontine network connectivity in parkinsonian patients with freezing of gait. Brain.

[5] Herman T, Weiss A, Brozgol M, Giladi N, Hausdorff JM (2014). Gait and balance in Parkinson’s disease subtypes: objective measures and classification considerations. J Neurol.

[6] de Laat KF, Tuladhar AM, van Norden AG, Norris DG, Zwiers MP, de Leeuw FE (2011). Loss of white matter integrity is associated with gait disorders in cerebral small vessel disease. Brain.

[7] Wang HC, Hsu JL, Leemans A (2012). Diffusion tensor imaging of vascular Parkinsonism: structural changes in cerebral white matter and the association with clinical severity. Arch Neurol.

[8] Seidler RD, Bernard JA, Burutolu TB, Fling BW, Gordon MT, Gwin JT (2010). Motor control and aging: links to age-related brain structural, functional, and biochemical effects. Neurosci Biobehav Rev.

[9] Jacobs JV, Lou JS, Kraakevik JA, Horak FB (2009). The supplementary motor area contributes to the timing of the anticipatory postural adjustment during step initiation in participants with and without Parkinson’s disease. Neuroscience.

[10] Cordo PJ, Nashner LM (1982). Properties of postural adjustments associated with rapid arm movements. J Neurophysiol.

[11] Nonnekes J, Snijders AH, Nutt JG, Deuschl G, Giladi N, Bloem B (2015). Freezing of gait: a practical approach to management. Lancet Neurol.

[12] Armstrong, RA. Visual symptoms in Parkinson’s disease. Parkinson Dis. 2011;2011:908306. 10.4061/2011/908306 PMC3109513 21687773

